# Chemical Composition and Biological Activity of *Hypericum* Species—*H. hirsutum*, *H. barbatum*, *H. rochelii*

**DOI:** 10.3390/plants13202905

**Published:** 2024-10-17

**Authors:** Jovan Baljak, Mirjana Bogavac, Maja Karaman, Branislava Srđenović Čonić, Biljana Vučković, Goran Anačkov, Nebojša Kladar

**Affiliations:** 1 Department of Pharmacy, Faculty of Medicine, University of Novi Sad, Hajduk Veljkova 3, 21000 Novi Sad, Serbia; jovan.baljak@mf.uns.ac.rs (J.B.); branislava.srdjenovic-conic@mf.uns.ac.rs (B.S.Č.); nebojsa.kladar@mf.uns.ac.rs (N.K.); 2 Center for Medical and Pharmaceutical Investigations and Quality Control, Faculty of Medicine, University of Novi Sad, Hajduk Veljkova 3, 21000 Novi Sad, Serbia; 3 Clinical Center of Vojvodina, Department of Obstetrics and Gynecology, Faculty of Medicine, University of Novi Sad, Hajduk Veljkova 3, 21000 Novi Sad, Serbia; 4 Department of Biology and Ecology, Faculty of Sciences, University of Novi Sad, Trg Dositeja Obradovica 2, 21000 Novi Sad, Serbia; maja.karaman@dbe.uns.ac.rs (M.K.); goran.anackov@dbe.uns.ac.rs (G.A.); 5 Department of Pathophysiology and Laboratory Medicine, Faculty of Medicine, University of Novi Sad, Hajduk Veljkova 3, 21000 Novi Sad, Serbia; biljana.vuckovic@mf.uns.ac.rs

**Keywords:** *Hypericum*, monoamine oxidase A, α-glucosidase, antimicrobial, antioxidants

## Abstract

St. John’s wort (*Hypericum perforatum*, Hypericaceae) is the most well-known species in the genus *Hypericum*, which comprises several hundred species. This study investigates the biological and phytochemical potential of the under-researched *Hypericum* species, *H. hirsutum*, *H. barbatum*, and *H. rochelii*. A high level of similarity between the chemical profiles of *H. hirsutum* and *H. barbatum* and the official source of the herbal drug (*H. perforatum*) was shown, but a higher content of quercetin and rutin was also found in all three evaluated species (116–230 µg/g dry herb). The highest amount of phenolics (195 mg GAE/g) was recorded in *H. hirsutum* extract, while the highest amount of flavonoids (47 mg QE/g) was recorded in *H. barbatum* extract. The evaluated species were excellent scavengers of DPPH, OH, and NO radicals, as well as strong ferric ion reducers in the FRAP test. Prominent monoamine oxidase A and α-glucosidase inhibition was observed, compared to modest inhibition of monoamine oxidase B, α-amylase, and acetylcholinesterase. High activity against Gram-positive MRSA *S. aureus* was demonstrated for the tested species, with MIC/MBC values recorded at 12.5 µg/mL. Antifungal activity against *Candida* strains was not observed. The obtained results emphasize the need for further investigation of species of the genus *Hypericum* to discover potentially new sources of biologically active compounds.

## 1. Introduction

St. John’s wort (*Hypericum perforatum*, Hypericaceae) is the most recognizable and extensively researched species of the genus *Hypericum*. It contains numerous secondary metabolites belonging to diverse chemical families, known for their biological activities [1]. Some of these compounds, such as naphthodianthrones (hypericin and pseudohypericin), phloroglucinols (hyperforin and adhyperforin), flavonoids and their glycosides (quercetin, rutin, quercitrin, hyperoside), as well as biflavonoids (amentoflavone), have attracted great research interest in recent years [2]. However, it is interesting that no currently available scientific consensus exists in term of compounds being responsible for recorded therapeutic effects, but most of the studies conclude that the reported health beneficial effects are a result of joint activity of secondary metabolites present in *H*. *perforatum* extracts [3]. The well-defined pharmacological potential of *H. perforatum* has suggested the application of water-alcoholic extract in treatment of mild to moderate forms of depression. Another, traditional but clinically effective, is usage of St. John’s wort oil macerate externally in the treatment of wounds, bruises, and eczema, or internally for treatment of various lesions of the gastrointestinal tract [4]. Also, other genus representatives are traditionally used in the ethnomedicine of different cultures [5].

The available results indicate that many other *Hypericum* species not only share the chemical profile of *H. perforatum*, but also contain higher amounts of previously identified secondary metabolites of interest [6,7]. This suggests a research direction aimed at their biological potential evaluation in order to discover new sources of biologically active compounds and potentially provide clinical significance. These results indicate various biological activities, such as antioxidant [8], antimicrobial [5], antidepressant [9], cytotoxic, and anti-inflammatory effects [10], but data are generally lacking for a large number of genus representatives [11]. In the recently published article of our research group, the biological activity of several *Hypericum* species has been extensively studied, revealing their significant antioxidant and antimicrobial potential. Namely, *H*. *triquetrifolium*, *H. maculatum*, and *H. tetrapterum* chemical profiling suggested the presence of a range of bioactive compounds, including hypericins, flavonoids, and phenolic acids, which contribute to wide spectra of pharmacological effects [12]. Following the same research direction, three so far less explored *Hypericum* species were analyzed in the current study. Specifically, *Hypericum rochelii* is distributed in the stony and calcareous habitats of southeastern Serbia and the Carpathians at an altitude between 600 and 1100 m [6]. *H. barbatum* can be found throughout the Mediterranean region in beech forests and serpentine rocks, while *H. hirsutum* is distributed in the wooded valleys and slopes of Europe and Western Siberia [5,13].

Antimicrobial resistance is a significant global health challenge that implicates the need for alternative treatments, with plant extracts emerging as desirable sources of potential antimicrobial agents [14]. *H. rochelii*, *H. hirsutum*, and *H. barbatum* extracts show outstanding antibacterial activity, especially against Gram-positive bacteria using laboratory strains [5]. The use of clinical bacterial strains to assess the antibacterial activities of plant extracts is essential for validating the relevance and potential application in clinical practice [15].

Therefore, the aim of this research was to evaluate the biological activities of ethanolic extracts of so far not extensively studied *Hypericum* species—*H. hirsutum*, *H. rochelii,* and *H. barbatum*. This included the assessment of antioxidant and antihyperglycemic potential, as well as potential to inhibit monoamine oxidase A and B (MAO-A and MAO-B) and acetylcholine esterase (AChE). Furthermore, chemical characterization of analyzed extracts in term of total phenolics and flavonoids content, as well as quantities of hypericin, hyperforin, and selected phenolic acids and flavonoids was performed.

## 2. Results and Discussion

### 2.1. Chemical Characterization of Hypericum Extracts

The amounts of total phenolics and total flavonoids in the studied extracts ranged from 139.14 to 195.30 mg GAE/g dried extract (de) and 29.32 to 46.98 mg QE/g de, respectively. The recorded values are consistent with previous analyses of these species [16,17,18,19,20] and analyses of *H. perforatum* extracts, but it is necessary to emphasize that for most of the examined taxa, this is one of the first comprehensive studies on the quantification of total phenolic components. The highest amount of phenolics was recorded in *H. hirsutum*, while the highest amount of flavonoids was recorded in *H. barbatum* extract. A comprehensive chemical profile analysis of the obtained extracts showed the abundance of several classes of compounds (Table 1).

Hypericin, an important chemotaxonomic marker of the genus *Hypericum,* was detected in all studied samples, although high abundance was characteristic for *H. barbatum* and *H. rochelii*, while a significantly lower amount was quantified in *H. hirsutum*. The hypericin content found in *H. barbatum* corresponded to previously published papers [21] or was somewhat higher than in the research conducted by Šmelcerović et al. [22]. Also, the level found in *H. hirsutum* was partially in agreement with previous results [21,23] or slightly higher than in the research published by Šmelcerović et al. [22] and Sagratini et al. [17]. Hyperforin was absent from *H. hirsutum* samples, which is a previously reported characteristic of that taxon [23]. Similarly, as in the case of hypericin, *H. barbatum* and *H. rochelii* also contained high quantity of hyperforin. Specifically, the *H. barbatum* hyperforin content was higher than reported in previous studies [17,22,23], which could be a result of differences in the applied extraction procedures. A taxon particularly rich in quercetin was *H. hirsutum*, while lower content was observed in the other investigated species. *H. hirsutum* quercetin content corresponded to previously published results, or was lower (2.58 mg/g de) [23]. However, although showing low abundance of quercetin, it is interesting that *H. barbatum* contained a high level of rutin—a quercetin glycoside, suggesting that *H. barbatum* accumulates more glycosidic forms. The amounts of rutin and quercetin found in *H. hirsutum* and *H. rochelii* were in agreement with the results of previous research [24]. Apigenin was not detected in any sample, which confirms the instability of the synthesis of this secondary metabolite, as well as its ability to dimerize in certain species of the genus *Hypericum* [25]. On the other hand, a particularly high content of amentoflavone was found in *H. barbatum* and *H. hirsutum*, while *H. rochelii* contained a lower amount of this biflavonoid. The abundance of amentoflavone in extracts of *H. hirsutum* corresponded to previously published research [24].

Considering the results of chemical characterization obtained for the evaluated species, it is possible to conclude that *H. hirsutum* and *H. barbatum* show a significant similarity in chemical profile with the official source of the herbal drug (*H. perforatum*). On the other hand, all three species evaluated in the current study are characterized by a higher content of quercetin and rutin [26,27].

### 2.2. Biological Potential of Evaluated Hypericum Species

#### 2.2.1. Antioxidant Potential

Recent studies have recognized plants and herbal preparations as important sources of antioxidants. This has encouraged their use in the prevention and treatment of various health disorders and as functional compounds in food and cosmetic products. Considering the complex dynamics of oxidative processes and the diverse characteristics of free radicals, it is essential to apply a variety of antioxidant assays in order to comprehensively evaluate the antioxidant properties of the substances [28]. Moreover, the mutual comparison of antioxidant potential results reported by different research groups is not a simple task. This is mainly a consequence of inconsistency in the actual experimental protocols applied. Specifically, it is highly probable that in the “same” assays, based on the identical chemical mechanism but implemented in different laboratories, variable concentrations of free radicals are generated, which directly affects the recorded antioxidant potential of evaluated agents. Therefore, in order to comprehensively evaluate the antioxidant properties of the examined *Hypericum* species, five antioxidant test-systems were applied in the current study, while the quality of the obtained results and relevance of conclusions regarding utilization potential were improved by providing results of the antioxidant potential of approved antioxidants—positive controls.

Previous research indicates that antioxidant potential is related to the presence of phenolic acids and flavonoids. Tested extracts showed strong antioxidant potential in the DPPH test system, with an RSC_50_ of 2.8–3.6 µg/mL (Table 2). *H. hirsutum* extracts were the most potent antioxidants, potentially due to the presence of chlorogenic acid, which is considered responsible for the neutralization of DPPH radicals. The obtained results are in line with previously published studies [21] and, although somewhat higher than the RSC_50_ value of the positive controls (quercetin dihydrate (QDH) and propyl gallate (PG)), still reasonably comparable. Hydroxyl radicals are known as reactive species that can be neutralized only by non-enzymatic antioxidants of endogenous or exogenous origin. The evaluated extracts neutralized OH radicals with RSC_50_ values ranging 49–59 µg/mL in the test system aimed at studying extracts’ protective effect toward carbohydrates. Although all extracts demonstrated similar OH^•^ neutralization potential, the extracts of *H. barbatum* were the most effective. However, the results were of modest to moderate relevance when compared to the positive controls. Moreover, the application of similar test systems evaluating the protective effects of extracts in the case of lipid substrates indicated even lower antioxidant potential. Specifically, the RSC_50_ values of the examined extracts ranged 383–410 µg/mL, which was significantly higher when compared to the positive control (BHT, RSC_50_ = 7.92 µg/mL).

Nitric oxide (NO) binding to a protein containing heme, iron, or copper, results in NO oxidation or reduction, thus producing highly reactive free radicals. All of the evaluated extracts were good scavengers of NO^•^ with RSC_50_ values ranging 21.69–33.64 µg/mL, especially when compared to the NO^•^ neutralization potential of propyl gallate (RSC_50_ = 8.90 µg/mL), whereas *H. rochelii* extract was the most effective antioxidant. The results of the FRAP test emphasized strong antioxidant potential of evaluated extracts since the obtained results ranged 142–160 mg AAE/g de [11]. *H. rochelii* demonstrated significant antioxidant activity, which is consistent with the study of Babota et al., where this effect was attributed to the high amount of phenolic acids. Moreover, studies have shown that the antioxidant activity of *Hypericum* species generally correlates with their total phenolics content [19].

#### 2.2.2. Inhibition of Biologically Important Enzymes

##### Inhibition of Acetylcholinesterase and Monoamine Oxidases A and B

St. John’s wort extracts are traditionally used for various neurological conditions, such as anxiety and depression, which are closely related to Alzheimer’s disease due to cognitive dysfunction. Acetylcholinesterase inhibition alleviates depressive episodes with a positive effect on symptoms in patients with Alzheimer’s disease. The study results indicate modest anticholinesterase activity of evaluated extracts when compared to galantamine, which is similar to previous conclusions regarding other *Hypericum* species [12]. The strongest anticholinesterase activity was recorded for *H. hirsutum* extracts (IC_50_ = 715.49 µg/mL) [18].

Inhibition of monoamine oxidase A (MAO-A) is a recognized pharmacological mechanism for alleviating symptoms of depression and anxiety, while MAO-B inhibition reduces the intensity of oxidative neurodegeneration in Parkinson’s disease. The evaluated extracts inhibited 50% of MAO-A activity in concentrations ranging 5–7.5 µg/mL. *H. hirsutum* extracts demonstrated the strongest anti-MAO-A potential, which can potentially be attributed to the high content of quercetin, which has been proven to selectively inhibit MAO-A [29]. Although lower anti-MAO-A potential of extracts is evident, when compared to moclobemide (positive control) and quercetin [30], it can be considered as comparable to the inhibition potential of the studied positive control, especially when bearing in mind that a comparison of the activity between a complex mixture of compounds (such as extracts) and a pure substance is being performed. On the other hand, the obtained results indicate significantly lower anti-MAO-B potential of the evaluated extract (IC_50_ values ranging 40–60 µg/mL), especially when compared to selegiline (IC_50_ = 0.22 µg/mL). Generally, the studied species showed higher MAO-A inhibition potential compared to *H. perforatum* [31], but the same order of magnitude of anti-MAO potential could be expected if taking into consideration the previous studies performed on *H. perforatum* [32].

##### Antihyperglycemic Potential

Herbal preparations reducing glycemia after meals decrease oxidative stress levels and could be of importance in the prevention of diabetes or if applied as co-therapy to conventional diabetes treatment. Previous reports suggest that secondary metabolites of herbal origin have the potential to inhibit α-amylase and α-glucosidase activity, while some phenolic compounds lead to the inactivation of these enzymes by non-specific binding [33]. *H. perforatum* extracts showed significant antihyperglycemic effects in streptozotocin-induced diabetic rats, reducing blood glucose levels by 70–72% after two weeks of treatment [34]. Also, St. John’s wort extract exhibits antihyperglycemic and antidiabetic effects by regulating AMPK in the liver [35]. *H. perforatum* contains secondary metabolites that inhibit α-glucosidase activity, with biapigenin being identified as a novel potent inhibitor [33,36].

The IC_50_ values obtained for the evaluated extracts regarding inhibition of α-amylase activity are in agreement with the results of previous studies. The strongest α-amylase inhibitory potential was demonstrated by *H. hirsutum* extract (IC_50_ = 80 µg/mL). In contrast to the *H. barbatum* and *H. rochelii* extracts, which are modest α-amylase inhibitors, *H. hirsutum* extract can be highlighted as highly potent when compared to inhibitory activity of acarbose and previously reported results for *H. triquetrifolium* [37], hyperoside, and quercetin [38], thus suggesting further possibility of isolating the compounds responsible for this effect. On the other hand, all evaluated extracts demonstrated prominent α-glucosidase inhibitory activity stronger than acarbose (positive control). The currently evaluated species have shown a similar α-glucosidase inhibitory activity as previously reported for *H. scruglii*, *H. hircinum* [39], and *H. patulum* [40]. Some of the possible mechanisms supporting this finding could be the presence of glycosides in the extracts, which, due to their similarity with the substrates of the studied enzymes, enable competitive inhibition. Moreover, there is also a possibility that compounds formed in the gastrointestinal tract by hydrolysis of the constituents present in the herbal extracts exhibit an additional inhibitory effect on the studied enzymes [41].

#### 2.2.3. Chemometric Approach—Biological Potential

**Figure 1:**
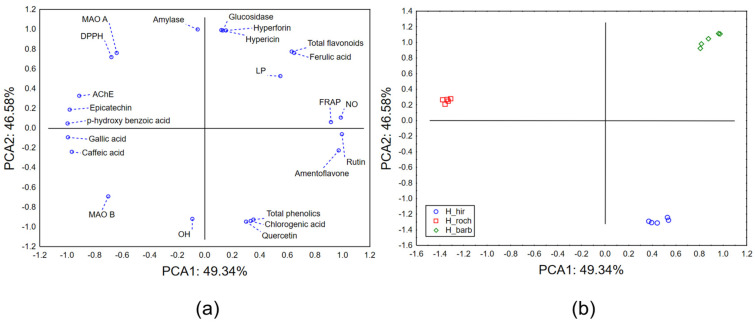
PCA-biological potential: (**a**) PCA loadings, (**b**) the position of the evaluated extracts in the space defined by the first two principal components (PCAs).

The Principal Component Analysis (PCA) applied on the dataset describing the biological potential and chemical profiles of the analyzed *Hypericum* species indicated that the first two principal components describe more than 95% of the samples’ variability. In terms of PCA1, most of the variability is described by quantified amounts of rutin, amentoflavone, epicatechin, p-hydroxy benzoic acid, gallic acid, caffeic acid, and ferric reduction antioxidant potential, as well as neutralization potential of NO radicals (Figure 1a). The shape of the variability in terms of PCA2 mostly correlated to the quantified amounts of hyperforin, hypericin, antihyperglycemic potential, and neutralization potential of OH radicals. The position of the evaluated extracts in the space defined by the first two principal components (Figure 1b) shows grouping of *H. rochelii* (H_roch) extracts in the negative part of the PCA1 as a result of the stronger neutralization potential of NO radicals, stronger ferric reduction antioxidant potential, and higher content of epicatechin and phenolic acids (p-hydroxy benzoic, gallic, and caffeic acid). The space defined by PCA2 indicates separative grouping of *H. hirsutum* (H_hir) extracts as a consequence of higher abundance of phenolic compounds (chlorogenic acid) and stronger antihyperglycemic potential. *H. barbatum* (H_barb) extracts are located in the positive part of PCA 1 since they contain higher amounts of total flavonoids, ferulic acid, and more prominent anti-MAO-B activity.

#### 2.2.4. Antibacterial and Anti-Candida Activity

The analyzed extracts demonstrated notable efficacy against all tested bacterial strains, while no antifungal activity was observed against *Candida* strains (Table 3). Particularly high activity was noted against Gram-positive MRSA *S. aureus*, with MIC/MBC values recorded at 12.5 µg/mL. This robust activity against MRSA is of particular importance given the clinical challenges associated with treating infections caused by this notoriously resistant pathogen [42]. Furthermore, all extracts displayed notable activity against other Gram-positive bacteria (*Enterococcus* sp.), albeit at slightly higher MIC/MBC values (25 µg/mL), thus indicating a broader spectrum of activity against this bacterial group. In terms of activity against Gram-negative bacteria, the extracts of significance were those obtained from *H. hirsutum* and *H. barbatum*. They exhibited the lowest MIC/MBC values, recorded at 12.5 µg/mL, against *P. mirabilis* and *P. aeruginosa*. Additionally, *P. vulgaris* displayed increased susceptibility to *H. barbatum* extract (MIC/MBC = 12.5 µg/mL). The antibiogram results published in our previous study [12] revealed that the *P. aeruginosa* isolate displayed multidrug resistance to commonly used antibiotics, highlighting the significance of high susceptibility of this bacterium to the *H. hirsutum* and *H. barbatum* extracts tested in the current study. The laboratory strain of *E. coli* demonstrated the lowest susceptibility to the tested extracts. Interestingly, the most effective extract against this strain was the *H. barbatum* extract, with a MIC of 25 µg/mL. *H. barbatum* extract in general had the superior antibacterial effect compared to other two plant extracts since four bacterial strains showed high susceptibility, with MIC/MBC of 12 µg/mL.

Numerous studies have investigated the antimicrobial properties of various *Hypericum* species extracts against different bacterial strains [5,6,12,43]. A recently published paper suggests that *H. rochelii* water-ethanolic extract shows notably lower efficacy against Gram-positive bacteria (particularly *S. aureus*) when compared to our extracts since reported MIC and MBC values ranged from 250 to 1000 mg/L and 500 to 2000 mg/L, respectively [6]. The current study findings also highlight greater efficacy of the analyzed extracts against both Gram-positive and Gram-negative bacteria compared to our previous research, where the other three *Hypericum* species were analyzed [12]. However, our findings are in agreement with results of Radulović et al. [43], who suggested significant antibacterial activity of *H. barbatum* and *H. hirsutum* methanolic extracts, whereas special emphasis was placed on *H. hirsutum*. On the other hand, extracts of *H. rochelii* and *H. hirsutum* from Bulgaria demonstrated lower MICs (ranging from 0.625 to 78 mg/L) against *S. aureus* and other Gram-positive bacteria [5] when compared to our results.

The higher antibacterial activity of *Hypericum* extracts, especially *H. hirsutum* and *H. barbatum,* recorded in our research could be explained by higher polarity of solvents used for extraction. Namely, phenolic compounds that are more polar and demonstrate high antibacterial activity, such as flavonoid glycosides, often cannot be fully extracted using solely organic solvents [44]. In the case of *H. hirsutum*, the synergistic effect of the detected flavonoids (amentoflavone, quercetin, and rutin) could be responsible for the recorded antibacterial activity, especially against *S. aureus*, since all these compounds were identified as highly potent inhibitors of MRSA [45,46]. Conversely, within the extracts of *H. barbatum* and *H. rochelii*, hypericin, a polycyclic phenol, and hyperforin, a prenylated derivative of phloroglucinol, might contribute to a high level of antibacterial activity against Gram-positive bacteria [12,47]. However, the *H. barbatum* extract is distinguished by its higher levels of amentoflavone, rutin, and ferulic acid, which, if acting synergistically with hyperforin and hypericin, could potentially enhance the antibacterial effectiveness of the extract. The available literature also implies that interaction with the membrane could serve as a crucial mechanism underlying the antibacterial activity of flavonoids, while some suggest that increased lipophilicity in flavonoids could contribute to an intensified interaction with the membrane, thereby potentially enhancing their antibacterial efficacy [48]. The superior activity of *H. hirsutum* and *H. barbatum* extracts against challenging pathogens such as *S. aureus, P. mirabilis,* and *P. aeruginosa* adds valuable insights to the existing knowledge on natural antimicrobial agents and underscores the importance of further exploration into their mechanisms of action and potential clinical applications in combating resistant bacterial infections.

## 3. Materials and Methods

### 3.1. Herbal Material and Preparation of Extracts

The studied herbal material consisted of the upper aerial parts of *H. barbatum* from Devetak Mountain (Republic of Srpska, Bosnia and Herzegovina), *H. hirsutum* from Mučanj Mountain (Serbia), and *H. rochelii* from Gornjak Gorge (Serbia) collected during the blooming stage. Voucher specimens (2-0400, 2-0414, 2-0664, respectively) were deposited in the BUNS Herbarium (Herbarium of the Department of Biology and Ecology, Faculty of Natural Sciences and Mathematics, University of Novi Sad). Dried herbal material (5 g) was grounded (sieve 355 µm) and subsequently extracted by maceration technique with 70% ethanol (m/m) for 72 h at room temperature (ration drug:solvent = 1:5), as suggested by the EMA, as well as *European Pharmacopoeia*, 6th Edition [49]. The obtained liquid extracts were filtered and evaporated to dryness using rotary evaporator (Rotavapor R-100, BÜCHI Labortechnik AG, Flawil, Switzerland). In order to study biological potential, we have dissolved dry extracts in distilled water in concentration 10% (m/m), whereas for chemical characterization dry extracts were dissolved in methanol (50%, m/m).

### 3.2. Chemical Profiling of Plant Extracts

The quantity of total phenolics in the studied extracts was determined spectrophotometrically, according to previously described procedure [50], while the obtained results were presented as mg of gallic acid equivalents (GAE) per g of dry extract (mg GAE/g de), based on a calibration curve obtained for gallic acid. Similarly, the total flavonoids were quantified according to the previously described method utilizing aluminum chloride as complexation reagent [50], whereas the concentration was expressed in mg of quercetin equivalents (QE) per g of dry extract (mg QE/g de). Two previously reported and validated high pressure liquid chromatography-based (HPLC-DAD) methods have been applied for detailed chemical profiling of the prepared extracts. An Agilent HP 1100 instrument (Agilent, Waldbronn, Germany) was utilized for analysis. Method 1, reported by Bradić et al. [51], was used for the quantification of hypericin and hyperforin. The compounds of interest were separated at 25 °C on Zorbax CB-C18 column (4.6 × 150 mm, 5 µm particle), while 10 µL of extract was injected. Method II was developed in accordance to a report of Ziaková et al. [52] and was used for quantification of rutin, quercetin, and gallic, chlorogenic, caffeic, and p-hydroxybenzoic acids. Briefly, gradient elution was applied (3.25 min, 0% B; 8 min, 12% B, 15 min, 25% B, 15.8 min, 30% B, 25 min, 90% B, and 25.4 min, 100% B) with the flow rate of 1 mL/min, where solvent A was 0.1% (*v*/*v*) solution of acetic acid in water and solvent B was 0.1% (*v*/*v*) solution of acetic acid in acetonitrile. The compounds were separated on Nucleosil C18 (4.6 × 250 mm, 4.6 µm particle) column heated at 30 °C, while 10 µL of extract was analyzed. The content of quantified secondary metabolites was expressed as µg/g of dry herbal material.

### 3.3. Antioxidant Potential Evaluation

#### 3.3.1. Free Radical Scavenging Capacity (RSC)

Free radical scavenging potential of the evaluated extracts was tested in vitro against 2,2-diphenyl-l-picrylhydrazyl (DPPH), hydroxyl (OH) and nitric oxide (NO) radicals, following the previously described procedures Specifically, the addition of extracts in different concentrations to the DPPH radical solution (final concentration in reaction mixture, c = 25 µM) and monitoring the reduction of absorbance at 515 nm was the working principle of applied DPPH test [12]. Furthermore, the neutralization potential regarding OH radicals was assayed through monitoring the degradation level of 2-deoxy-D-ribose by the OH radicals generated in the Fenton’s reaction (final reaction mixture c(OH^•^) = 0.7 mM). Namely, malondialdehyde (MDA), as the resulting degradation product reacts with thiobarbituric acid (TBA) by forming a complex showing absorption maximum at 532 nm. Another model of OH radical neutralization potential, which simulates the protection of lipids, was applied [12]. Briefly, liposome emulsion was used as source of lipids subjected to oxidative degradation by OH radicals. As in the previously described model, lipid degradation leads to formation of MDA, which is a TBA reactive substance, and changes in its levels are monitored at 532 nm. The potential of the evaluated extracts to neutralize NO radicals (generated from sodium nitroprusside; final reaction mixture c(NO^•^) = 3.5 mM) was estimated through reduction in absorbance at 546 nm after the addition of Griess’s agent, which reacts with free NO radicals by forming a purple-colored complex. In order to obtain realistic insight into the obtained results of antioxidant potential, we have evaluated under the same experimental conditions positive controls—recognized antioxidants, such as propyl gallate (PG), quercetin dihydrate (QDH), ascorbic acid (AA), and butylated hydroxytoluene (BHT).

The percentage of neutralization of the tested free radicals was calculated using the following Equation (1):

RSC (%) = 100 × (A_blank_ − A_sample_/A_blank_) (1)

where A_blank_ and A_sample_ are absorbances of reaction mixtures containing no added extract and increasing concentrations of evaluated extracts, respectively. This enabled us to apply regression analysis and obtain fitting equation describing dependence of radical scavenging capacity from extract concentration, which was applied for calculation of extract concentrations exhibiting 50% of free radicals neutralization (RSC_50_).

#### 3.3.2. Ferric Reduction Antioxidant Potential (FRAP)

The Fe^3+^ reduction ability of extracts was measured colorimetrically according to Lesjak et al. [50]. Namely, the resulting Fe^2+^ forms a blue-colored complex with 2,4,6-tripyridyl-S-triazine characterized by absorption maximum at 593 nm. The obtained results are expressed as mg of ascorbic acid equivalents per g of dry extract (mg AAE/g de), based on previously determined antioxidant potential of ascorbic acid.

### 3.4. Biologically Important Enzymes Inhibition

#### 3.4.1. Inhibition of Acetylcholinesterase

A modified Ellman’s method was applied for studying anticholinesterase activity of extracts [50]. The reaction mixture contained phosphate buffer (pH = 7.2), 5,5’-dithiobis-(2-nitrobenzoic acid)-DTNB as color indicator and acetylcholinesterase solution (8.15 U/L was the final reaction activity) in which the increasing concentration of extracts was added and incubated for 15 min at room temperature. Subsequent addition of acetylthiocholine iodide (substrate) was followed by monitoring the change of absorbance at 405 nm for 3 min. The test control mixture contained distilled water instead of an extract and was considered as 100% of enzyme activity, while galantamine was used as positive control.

#### 3.4.2. Monoamine Oxidase A (MAO-A) and Monoamine Oxidase B (MAO-B) Inhibition

In accordance with the study published by Samoylenko [53], the inhibition potential of extracts regarding MAO-A and MAO-B (human recombinant) was determined. The reaction mixtures contained the corresponding enzyme (MAO-A or MAO-B), phosphate buffer, kynuramine (substrate), as well as increasing concentrations of extracts. The occurrence of 4-hydroxyquinoline after enzymatic degradation of kynurenine was monitored spectrofluorimetrically. The final MAO-A and MAO-B enzyme concentrations in the reaction mixtures were 5 μg/mL and 10 μg/mL, respectively. The phosphate buffer (instead of the extract) was added to the reaction mixture considered as control (100% of enzyme activity), while moclobemide and selegiline were used as positive controls for MAO-A and MAO-B inhibition, respectively.

#### 3.4.3. Inhibition of α-Amylase and α-Glucosidase

The anti-α-amylase potential was estimated spectrophotometrically following the procedure previously reported by Kladar et al. [12]. Increasing concentrations of the extracts were incubated at room temperature with the mixture of porcine α-amylase (final reaction mixture activity 0.6 U/mL), Starch azure^®^ (Sigma Aldrich, St. Louis, MO, USA), and sodium phosphate buffer (pH = 7.2). After 10 min the reaction was stopped as a result of acetic acid (50%, m/m) addition. Test control solutions contained distilled water instead of studied extract, while acarbose was used as positive control.

Similarly, the anti-α-glucosidase (isolated from *Saccharomyces cerevisiae*) activity of tested extracts was studied. The reaction mixture contained potassium phosphate buffer (pH = 6.8), glutathione solution (reduced form), α-glucosidase (final activity in the reaction mixture was 7.6 U/L), p-nitrophenyl-α-D-glucoside (PNP-Gluc) as substrate, and increasing concentrations of tested extracts. After incubation period (37 °C for 20 min), the reaction was stopped by adding Na_2_CO_3_ solution. The test control solution contained distilled water instead of the herbal extracts, while positive control was acarbose.

The percentage of evaluated enzyme inhibition was calculated according to the following Equation (2):

I (%) = 100 − (A_sample_/A_control_) × 100 (2)

where A_sample_ was the absorbance of the reaction mixture containing tested extract and A_control_ was the absorbance of the test control mixture containing no extract and being considered as 100% of enzyme activity.

The results of enzyme inhibitory potential recorded at different concentration levels were analyzed by regression approach and fitted in order to obtain the equation of fitting curve describing inhibitory potential dependence from concentration. This enabled us to calculate extract concentrations required for inhibiting 50% of enzyme activity (IC_50_).

### 3.5. Antimicrobial Activity

The antimicrobial activity was assessed by double micro-dilution method in order to determine the minimum inhibitory concentration (MIC) and bactericidal/fungal concentration (MBC/MFC) against six clinical bacterial strains and two Candida strains isolated from pregnant women displaying symptoms of vaginal infections. This experiment was conducted according to Clinical and Laboratory Standards Institute (CLSI) procedure and previously established protocols [54,55]. Six bacterial strains, including two Gram-positive (*Enterococcus* sp., *S. aureus* MRSA) and four Gram-negative strains (Escherichia coli, Pseudomonas aeruginosa, Proteus mirabilis, Proteus vulgaris) were used. Additionally, antifungal activity was tested against *C. albicans* strains. Two laboratory strains were sourced from the Department of Biology and Ecology, Faculty of Sciences (University of Novi Sad), while four clinical isolates were collected during routine gynecological examinations of women and obtained from the Department of Obstetrics and Gynecology—Clinical Center of Vojvodina. The utilization of these strains was approved by the Faculty of Medicine Novi Sad Ethics Committee. Microtiter plates were then incubated for 24 h at 37 °C, after which MIC and MBC values were determined.

### 3.6. Data Processing

The obtained results were processed using Microsoft Office Excel (v2019) and Tibco Statistica (v13.5). The results were analyzed by application of descriptive statistics, as well as by univariate and multivariate analysis (principal components analysis—PCA). The statistical significance of differences between the species was analyzed by Kruskal–Wallis ANOVA and subsequent multiple comparisons of mean ranks.

## 4. Conclusions

This research showed a high level of similarity in the chemical profiles of *H. hirsutum* and *H. barbatum* with *H. perforatum*. All three evaluated species proved to be good scavengers of free radicals and also to contain a higher amount of quercetin and rutin. A strong inhibition of monoamine oxidase A and α-glucosidase was observed, which indicates potential application in treatment of depression and type 2 diabetes mellitus. All analyzed *Hypericum* species were of great potential for the treatment of infections caused by Gram-positive bacteria, while of special importance was the effect against MRSA *S. aureus*. The obtained results highlight the importance of conducting further in vivo preclinical and clinical studies on the evaluated *Hypericum* species in order to clarify the clinical significance and safety of their potential application.

## Figures and Tables

**Table 1:** Comprehensive chemical profile of evaluated species.

Sample		*H. hirsutum*	*H. rochelii*	*H. barbatum*
Variables				
Total phenolics (mg GAE/g de)		195.30 (6.74) ^a^	137.50 (4.22) ^b^	139.14 (5.98) ^b^
Total flavonoids (mg QE/g de)		29.32 (1.35) ^a^	29.76 (1.54) ^a^	46.98 (2.11) ^b^
Dry extract yield (%)		19.76 (1.11) ^a^	27.66(1.88) ^b^	17.37 (1.35) ^a^
Class of compounds	Compound	µg/g dry herb		
Naphthodianthrones	Hypericin	70.02 (3.32) ^a^	1044.53 (11.53) ^b^	1838.39 (45.15) ^c^
Phloroglucinols	Hyperforin	nd ^a^	1047.66 (21.76) ^b^	1993.18 (114.65) ^c^
Biflavonoids	Amentoflavone	295.26 (2.82) ^a^	51.54 (0.73) ^b^	280.96 (7.56) ^c^
Flavonoids and flavonoid glycosides	Apigenin	nd ^a^	nd ^a^	nd ^a^
	Naringenin	682.22 (3.71) ^a^	nd ^b^	nd ^b^
	Rutin	278.12 (6.99) ^a^	133.72 (0.37) ^b^	301.61 (12.39) ^a^
	Quercetin	230.47 (2.95) ^a^	121.14 (5.72) ^b^	116.64 (4.32) ^c^
	Epicatechin	nd ^a^	386.70 (13.88) ^b^	nd ^a^
Phenolic acids	Ferulic acid	nd ^a^	nd ^a^	116.49 (0.26) ^b^
	Gallic acid	35.59 (1.31) ^a^	123.99 (1.78) ^b^	nd ^c^
	Chlorogenic acid	31.69 (0.84) ^a^	nd ^b^	nd ^b^
	Caffeic acid	37.10 (1.47) ^a^	66.04 (1.43) ^b^	17.00 (0.75) ^c^
	p-hydroxybenzoic acid	48.63 (1.97) ^a^	327,31 (10.37) ^b^	nd ^c^

The results are presented as an average value (standard deviation) (Xm (S.D.)) of three repeated measurements. nd—not detected. The different lower-case letters indicate statistically significant differences (*p* < 0.05).

**Table 2:** Biological potential of investigated *Hypericum* species.

Sample	*H. hirsutum*	*H. rochelii*	*H. barbatum*	Positive Control
Variable	RSC_50_ (µg/mL)			
DPPH	2.81 (0.03) ^a^	3.63 (0.01) ^b^	3.20 (0.09) ^c^	QDH, RSC_50_ = 1.08 (0.10) PG, RSC_50_ = 0.59 (0.02)
NO	29.90 (1.85) ^a^	21.69 (1.79) ^b^	33.64 (1.65) ^a^	PG, RSC_50_ = 8.90 (0.75)
OH, carbohydrate substrate *	59.29 (4.26) ^a^	53.25 (1.51) ^b^	49.77 (3.00) ^b^	BHT, IC_50_ = 0.04 (0.00) AA, IC_50_ = 2.26 (0.19) PG, IC_50_ = 10.15 (0.65)
OH, lipid substrate **	384.97 (2.36) ^a^	383.76 (7.40) ^a^	409.61 (4.80) ^b^	BHT, IC_50_ = 7.92 (0.66)
FRAP (mg AAE/g de)	155.82 (10.34) ^a^	142.31 (6.84) ^a,b^	160.89 (5.79) ^a^	/
Enzyme inhibition	IC_50_ (µg/mL)			Positive control
AChE	715.49 (38.44) ^a^	947.77 (49.17) ^b^	756.57 (26.54) ^a^	Galantamine IC_50_ = 9.11 (0.64)
MAO-A	5.11 (0.11) ^a^	8.69 (0.21) ^b^	7.51 (0.22) ^c^	Moclobemide IC_50_ = 0.71 (0.08)
MAO-B	60.18 (3.88) ^a^	61.76 (4.11) ^a^	40.50 (2.45) ^b^	Selegiline IC_50_ = 0.22 (0.02)
α-amylase	80.45 (3.65) ^a^	977.93 (38.99) ^b^	1343.55 (48.55) ^c^	Acarbose IC_50_ = 5.35 (0.72)
α-glucosidase	13.08 (0.25) ^a^	17.10 (0.39) ^b^	20.03 (0.42) ^c^	Acarbose IC_50_ = 48.76 (3.45)

The results are presented as an average value (standard deviation) (Xm (S.D.)) of three repeated measurements. The different lower-case letters indicate statistically significant differences (*p* < 0.05). QDH—quercetin dihydrate, PG—propyl gallate, BHT—butylated hydroxytoluene, AA—ascorbic acid. * Monitoring the degradation of 2-deoxy-D-ribose by OH radicals generated in the Fenton reaction. ** Protection of lipids using liposome emulsion subjected to oxidative degradation by OH radicals.

**Table 3:** Antimicrobial activity of studied *Hypericum* extracts (values expressed in µg/mL).

Agent	*H. hirsutum*		*H. rochelii*		*H. barbatum*	
Microbe	MIC	MBC	MIC	MBC	MIC	MBC
*S. aureus* ^H^ MRSA	12.5	12.5	12.5	12.5	12.5	12.5
*E. coli* ^L^	50	50	50	100	25	50
*P. mirabilis* ^H^	12.5	12.5	25	25	12.5	12.5
*P. aeruginosa* ^H^	12.5	12.5	25	25	12.5	12.5
*Enterococcus* sp. ^L^	25	25	25	25	25	25
*P. vulgaris* ^L^	50	50	25	50	12.5	12.5
*Candida* ^L^	/	/	/	/	/	/
*Candida* ^H^	/	/	/	/	/	/

Legend: ^H^—human isolate, ^L^—laboratory strain.

## Data Availability

Data are contained within the article.
